# An Evidence-Based Review Literature About Risk Indicators and Management of Unknown-Origin Xerostomia 

**Published:** 2013-05

**Authors:** Farzaneh Agha-Hosseini, Mahdieh-Sadat Moosavi

**Affiliations:** 1 Professor, Dental Research Center, Dentistry Research Institute, Department of Oral Medicine, School of Dentistry, Tehran University of Medical Sciences, Tehran, Iran; 2 Assistant Professor, Department of Oral Medicine, School of Dentistry, Tehran University of Medical Sciences, Tehran, Iran

**Keywords:** Xerostomia; Etiology; Saliva

## Abstract

This evidence-based article reviews risk indicators and management of unknown-origin xerostomia.

Xerostomia and hyposalivation refer to different aspects of dry mouth. Xerostomia is a subjective sensation of dry mouth, whilst hyposalivation is defined as an objective assessment of reduced salivary flow rate. About 30% of the elderly (65 years and older) experience xerostomia and hyposalivation. Structural and functional factors, or both may lead to salivary gland dysfunction.

The EBM literature search was conducted by using the medical literature database MEDLINE via PubMed and OvidMedline search engines. Results were limited to English language articles (1965 to present) including clinical trials (CT), randomized controlled trials (RCT), systematic reviews and review articles. Case control or cohort studies were included for the etiology. Neuropathic etiology such as localized oral alteration of thermal sensations, saliva composition change (for example higher levels of K, Cl, Ca, IgA, amylase, calcium, PTH and cortisol), lower levels of estrogen and progesterone, smaller salivary gland size, and illnesses such as lichen planus, are risk indicators for unknown-origin xerostomia. The management is palliative and preventative. Management of symptoms includes drug administration (systemic secretogogues, saliva substitutes and bile secretion-stimulator), night guard, diet and habit modifications. Other managements may be indicated to treat adverse effects. Neuropathic etiology, saliva composition change, smaller salivary gland size, and illnesses such as oral lichen planus can be suggestive causes for unknown-origin xerostomia. However, longitudinal studies will be important to elucidate the causes of unknown-origin xerostomia.

## Introduction

Xerostomia is a subjective sensation of dry mouth [1], whilst hyposalivation is defined as an objective assessment of reduced salivary flow rate. However, a 50% reduction in salivary flow, especially unstimulated salivary Saliva has a critical role in the maintenance of oropharyngeal health. Saliva consists of water, proteins and electrolytes that improve taste, speech and swallowing, and facilitate irrigation, lubrication and protection of the mucous membranes in the upper digestive tract. Saliva also provides antimicrobial (by various antimicrobial components such as mucin, histatins, lysozyme and lactoferrin) and buffering activities that protect the teeth from dental caries. So xerostomia and salivary dysfunction can impair a person’s quality of life by producing oral and pharyngeal disorders [9, 11, 12]. The aim of this article is to discuss xerostomia with an unknown etiology from the aspects of risk indicators and management.

**Table 1:** Questionnaire Used for Selection of Subjects with Xerostomia

1 Does your mouth feel dry when eating a meal?
2 Do you have difficulties swallowing any food?
3 Do you need to sip liquids to aid in swallowing dry food?
4 Does the amount of saliva in your mouth seem to be reduced most of the time?
5 Does your mouth feel dry at night or on awakening?
6 Does your mouth feel dry during the daytime?
7 Do you chew gum or use candy to relieve oral dryness?
8 Do you usually wake up thirsty at night?
9 Do you have problems in tasting food?
10 Does your tongue burn?
**Response options: yes/no**

**Table 2:** The Xerostomia Inventory (XI)

I sip liquids to help swallow food
My mouth feels dry when eating a meal
I get up at night to drink
My mouth feels dry
I have difficulty in eating dry food
I suck sweets or cough lollies to relieve dry mouth
I have difficulties swallowing certain foods
The skin of my face feels dry
My eyes feel dry
My lips feel dry
The inside of my nose feels dry
Response options: never (scoring 1), hardly (2), occasionally (3), fairly often (4) and very often (5)

## Discussion

Evidence-based medicine (EBM) search procedures: The aim of EBM is to describe a clinical question and the information required to solve the problem.

This aim is achieved by conducting a well-organized search of the literature, selecting the best of the appropriate studies, applying rules of evidence to determine their relative validity, extracting the clinical points and applying them to the patients problem considerating the patient’s ideals and expectations [13].

Our question was: What are the risk indicators of xerostomia with unknown etiology, and what therapies can help to alleviate the patient's symptoms?

The EBM literature search was conducted by using medical literature database MEDLINE via PubMed and OvidMedline search engines. Individual key words, alone or in combination, included “xerostomia”, “dry mouth”, “etiology”, “management”, “therapy” and “treat”. Results were limited to English language articles (1965 to present) including clinical trials (CT), randomized controlled trials (RCT), systematic reviews and review articles. 

Case control or cohort studies were included for etiology. 

**Fig 1:**
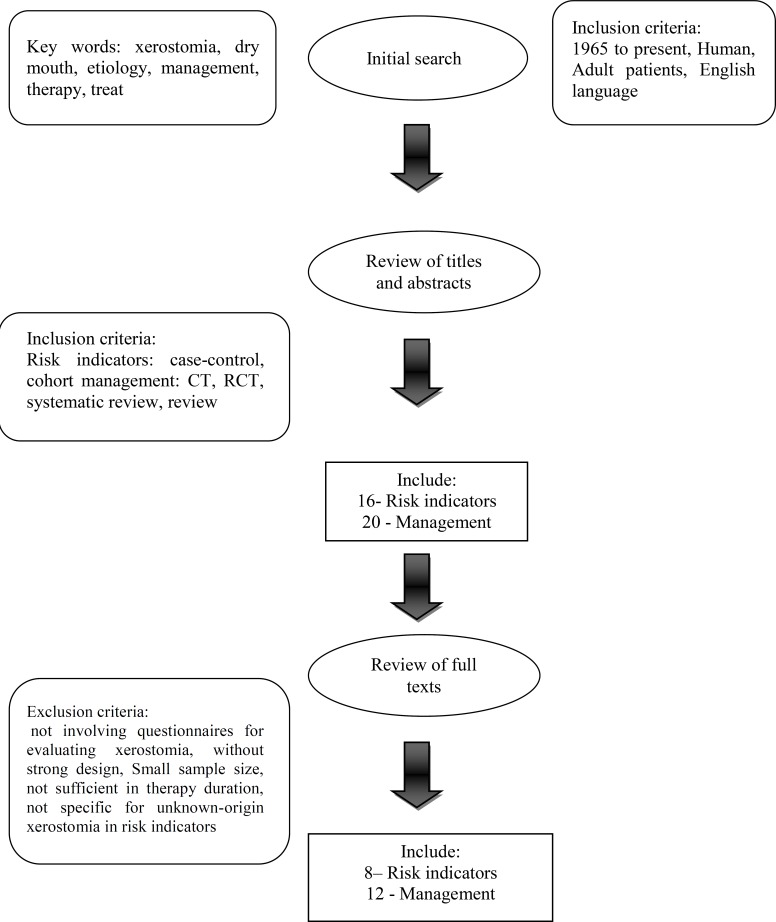
Search Strategy

As RCT would not be ethical for etiology, they were not expected to be found. The search strategy is shown in Figure 1, Table 3 and Table 4.

**Inclusion criteria**

- Studies including adult patients with xerostomia

**Exclusion criteria**

- Studies that did not involve questionnaires (or asking only one relative question) to evaluate subjective symptoms of xerostomia

**Table 3:** Rejection Table for Risk Indicators and Management

**Article**	**Reason for rejection**
Strietzel FP, Martín-Granizo R, Fedele S, Lo Russo L, Mignogna M, Reichart PA, Wolff A. Electrostimulating device in the management of xerostomia. Oral Dis. 2007;13(2):206-13.	Did not involve questionnaires for evaluating xerostomia, used verbal interview Results based on once using of therapeutic device. Treatment effect not large enough Small sample size,20
Bernardi R, Perin C, Becker FL, Ramos GZ, Gheno GZ, Lopes LR, Pires M, Barros HM. Effect of pilocarpine mouthwash on salivary flow. Braz J Med Biol Res. 2002;35(1):105-10.	Subjects were not xerostomic patients and treatment increased salivary flow in healthy volunteers Results based on once using of therapeutic mouthwash. Treatment effect not large enough Assessing stimulated salivary flow rate not unstimulated salivary flow rate that is more relevant to dry mouth feeling
Weiss WW Jr, Brenman HS, Katz P, Bennett JA. Use of an electronic stimulator for the treatment of dry mouth. J Oral Maxillofac Surg. 1986;44(11):845-50.	Did not involve questionnaires for evaluating xerostomia Without clear inclusion and exclusion criteria, especially for drug users No severity of xerostomia (subjective aspect of dry mouth) was examined before the treatment
Ami S, Wolff A. Implant-supported electrostimulating device to treat xerostomia: a preliminary study. Clin Implant Dent Relat Res. 2010;12(1):62-71.	Case report study
van den Berg I, Pijpe J, Vissink A. Salivary gland parameters and clinical data related to the underlying disorder in patients with persisting xerostomia. Eur J Oral Sci. 2007;115(2):97-102.	No controlled group for confounding factors, sialochemisty and sialography findings should compare with healthy subjects

**Risk Indicators**

Structural or functional factors, or both, may lead to salivary gland dysfunction. Structural salivary gland disorders include Sjo¨gren’s syndrome, sarcoidosis, post-irradiation damage, developmental anomalies, and diabetes mellitus. Chronic anxiety states, dehydration, and drug therapy are functional causes of xerostomia [14].

For a brief review, Table 4 lists the causes of xerostomia with known etiology [11, 15, 16].

Conditions which may be associated with unknown-origin xerostomia include:

***Neuropathic etiology:*** Patients with idiopathic oral sensorial complaints–burning mouth syndrome (BMS), dysgeusia (taste disturbances), and xerostomia (dry mouth) –were found to experience localized oral alteration of thermal sensations, expressed by higher warm and cold thresholds. 

They also had lower scores for tonic painful stimuli in the oral cavity. This profile of decreased sensation is usually seen in skin regions affected by either poly- or mono-neuropathy. Therefore, sensorial/taste analysis should be used in the routine diagnostic setup of patients with xerostomia, and therapeutic means aimed at correcting the disorders should be considered [17].

***Saliva composition change:*** There are significant alterations in the salivary composition of xerostomia patients. For example, higher levels of K, Cl, Ca, IgA, and amylase were found in these patients [17, 18] ; also estrogen and progesterone are significantly lower, and calcium, PTH and cortisol are higher in menopausal women with xerostomia compared with their control group6, [10, 19-21]. 

The hypothesis in these series of studies is that changes in the hormonal status of menopausal women result in a saliva composition change that can cause xerostomia. 

Therefore, the higher salivary level of PTH and cortisol in menopausal women with xerostomia suggests that they are at higher risk of bone mineral density loss, and we have recently found that menopausal women who lose bone mineral density may experience xerostomia [22]. However, in most cases, the reason for altered salivary composition is not yet known. 

***Smaller salivary gland size:*** The size of the parotid and submandibular glands, and the combined three major salivary glands of unknown-origin xerostomia patients were smaller than the controls. There were no pathological aspects to any of the magnetic resonance images (MRI) for the salivary glands of unknown-origin xerostomia patients. In spite of the small sample size of the case and control groups in this study, we included it because it is unique in kind, and has a strong design [4].

***Other illnesses:*** There is an association between oral lichen planus and xerostomia in some patients. But a possible pathogenesis of xerostomia in patients with OLP is not readily clear [14].

**MANAGEMENT**

Usually, in a few xerostomia patients, the clinician can identify the cause of xerostomia, so the management is palliative and preventative. For this reason management of xerostomia with or without known-origin etiology is the same. Management should be aimed to relieving symptoms, using preventive measures, treating oral conditions, increasing salivary function (if possible), and managing any underlying systemic conditions [2].

Complications of xerostomia include an increased rate of caries (particularly caries in cervical and cusps), a tendency toward acute gingivitis, dysphagia, dysgeusia, candidal infection (e.g. acute pseudomembranous candidiasis, angular cheilitis, median rhomboid glossitis, denture-associated stomatitis), burning tongue/depapillation of the tongue, and oral mucosal discomfort [16, 23].

**Table 4:** Acceptance Table for Risk Indicators and Management

**Article**	**Authors**	**Year**
Association between regional idiopathic neuropathy and salivary involvement as the possible mechanism for oral sensory complaint	Granot M and Nagler RM	2005
Sialochemical and gustatory analysis in patients with oral sensory complaints	Nagler RM and Hershkovic O	2004
Relationship of stimulated saliva 17 bet-aestradiol and oral dryness feeling in menopause	Agha Hosseini F et al.	2009
Stimulated and unstimulated saliva progesterone in menopausal women with oral dryness feeling	Mirzaii-Dizgah I and Agha-Hosseini F	2010
Serum and stimulated whole saliva parathyroid hormone in menopausal women with oral dry feeling	Agha Hosseini F et al.	2009
Relationship of stimulated whole saliva cortisol level with the severity of a feeling of dry mouth in menopausal women	Agha Hosseini F et al.	2010
Small salivary gland size in patients with xerostomia of unknown etiology	Ono K et al.	2009
An association between oral lichen planus and a persistently dry mouth	Colquhoun AN and Ferguson MM	2004
Diagnosis and treatment of xerostomia (dry mouth)	Napeñas JJ	2009
A neglected symptom	Sreebny LM et al.	1987
The oral mucosa as a therapeutic target for xerostomia	Thelin WR et al.	2008
Salivary gland dysfunction: a review of systemic therapies	Grisius MM	2001
Difficulties in dental prescribing of saliva substitutes for xerostomia	Frost PM	2002
A doubleblind, crossover study of Biote`ne Oralbalance and BioXtra systems as salivary substitutes in patients with post-radiotherapy xerostomia	Shahdad S.A et al.	2005
Efficacy of the BioXtra dry mouth care system in the treatment of radiotherapy- induced xerostomia	Dirix P et al	2007
Treatment of xerostomia with the bile secretion stimulating drug anethole trithione: a clinical trial	Hamada T et al.	1999
The application of a night guard for sleep-related xerostomia	Yamamoto K et al.	2008
Dry mouth and its effects on the oral health of elderly people	Turner MD and Ship JA	2008
An update of the etiology and management of xerostomia	Porter SR et al.	2004
Xerostomia: an update for clinicians	Hopcraft MS and Tan C	2010

Well-developed evidence-based management recommendations have the potential to enhance clinical practice, improve the quality of oral health care, lead to better patient outcomes, improve cost-effectiveness, and identify areas of further research needs [24]. This article briefly reviews treatment modalities that can be useful for unknown-origin xerostomia. However, professional judgment and patient preference may support a specific recommendation for an individual patient [24].

**Management of symptoms**

**Drugs**

***Systemic secretogogues:*** These can be useful for alleviating dryness symptoms and improving the protective effects of natural saliva. Pilocarpine and cevimeline are approved for the treatment of xerostomia. Pilocarpine is approved for Sjo¨gren’s syndrome and radiotherapy-induced xerostomia, and cevimeline for Sjo¨gren’s syndrome. 

The recommended dose is 30 mg three times daily for cevimeline, and 5-10 mg three times daily for pilocarpine taken orally. Both drugs have side effects, including sweating, rhinitis, increased pancreatic secretion, and urinary and gastrointestinal disturbances. Less regular and more serious adverse side effects of pilocarpine and cevimeline involve the cardiovascular and respiratory systems. The use of pilocarpine and cevimeline is contra-indicated in patients with gastric ulcer, uncontrolled asthma, or hypertension, or in patients on β-blockers [2, 16, 25, 26].

Saliva substitutes: These drugs attempt to replace the saliva, and with moistening properties, provide prolonged mucosal wetting. The most common substance is water but patients experience only temporary relief [2, 27]. Products include “artificial” salivas, rinses, gels, and sprays which may contain carboxymethylcellulose (CMC), a mucopolysaccharide glycerate polymer gel base, or natural mucins, singly or in combination. 

These products together have exhibited a mild effect on the subjective complaint of xerostomia, but no effect on objective measurements of hyposalivation [2]. 

There is a broad range of commercial products on the market. These products improve patient reported symptoms of xerostomia and the quality of life. Saliva Orthana spray TM is not acidic and contains fluoride and is safe for use in dentate patients and it contains porcine mucin. Products such as Biotene OralBalance are gel based. Patient preference dictates the most effective preparation [28, 29].

***Bile secretion-stimulating drug:*** Anethole trithione sufficiently stimulates salivation and improves xerostomia. The usual dose is 25 mg 3 times a day [26, 30]. 

**Night guard**

A night guard would be a helpful device for treatment, if sleep-related xerostomia was caused by abnormal masticatory muscle action.

**Table 5:** Causes of Xerostomia with Known Etiology

Drugs Local radiation Chemotherapy Chronic graft-versus-host disease Diseases of the salivary glands Sjo¨gren’s syndrome Sarcoidosis HIV disease Hepatitis C virus infection Primary biliary cirrhosis Cystic fibrosis Vasculitis Diabetes mellitus Renal dialysis *Rare causes* Amyloidosis Hemochromatosis Wegener’s disease Salivary gland agenesis (with or without ectodermal dysplasia) Triple A syndrome

Night guard can improve sleeping patterns for individuals with dry mouth, who are often awakened as a result of this condition. This improvement could be as a result of at least one of the following three factors: an increase of salivary secretion, the preservation of saliva volume in the oral cavity, or a decrease of saliva vaporization [31].

***Diet and habit modifications:*** These include frequent and regular sips of water, avoidance of dry, hard, sticky, acidic foods and avoidance of excess caffeine and alcohol [2].

**Other methods of stimulating salivation**

Chewing sugar-free, xylitol-containing mints, candies, and gum. These are salivary output stimulators [2, 9, 15]. Acupuncture: A systematic review in 2004 showed that there is no evidence for the efficacy of acupuncture in the management of xerostomia [31].

**Management of the adverse effects of xerostomia**

Increased frequency of oral/dental evaluation: to assess patients for oral complications of low salivary output [9, 23]. 

Fluoride application: Varnish (0.5% NaF) - daily use of fluoridated dentifrice, Topical - over-the-counter (0.05% NaF), prescription (1.0% NaF, 0.4% SnF). Daily use of neutral pH sodium fluoride is the most effective means of preventing rampant hyposalivation-induced caries [2].

Antifungal therapy for oral candidiasis: Reduction of saliva predisposes patients to an overgrowth of the fungus Candidia albicans. These are the recommendations for antifungal therapy:

- Chlorhexidine (CHX) 0.12%: rinse, swish, and spit 10 ml twice per day
- Antifungal rinses: nystatin oral suspension (100,000 units/milliliter), rinse four times per day
- Antifungal ointments: nystatin ointment applied 4 times per day
- Clotrimazole troches: 10 mg dissolved orally 4-5 times daily for 10 days
- Systemic therapy for immunocompromised patients
- Denture antifungal treatment: soaking of denture for 30 min daily in benzoic acid, 0.12% chlorhexidine or 1% sodium hypochlorite [2, 9, 11].

## CONCLUSION

Neuropathic etiology, saliva composition change, smaller salivary gland size, and illnesses such as oral lichen planus can be suggestive causes for unknown-origin xerostomia in a menopausal woman. However, longitudinal studies will be important to elucidate the causes of unknown-origin xerostomia. Management of patients with this kind of xerostomia is palliative and preventative.
